# *Faecalibacterium prausnitzii* Inhibits Interleukin-17 to Ameliorate Colorectal Colitis in Rats

**DOI:** 10.1371/journal.pone.0109146

**Published:** 2014-10-02

**Authors:** Mingming Zhang, Xinyun Qiu, Hao Zhang, Xiaotong Yang, Na Hong, Yonghua Yang, Hui Chen, Chenggong Yu

**Affiliations:** 1 Department of Gastroenterology, Nanjing Drum Tower Hospital, the Affiliated Hospital of Nanjing University Medical School, Nanjing, China; 2 Department of Digestive Diseases, Huashan Hospital, Fudan University, Shanghai, China; 3 State Key Laboratory of Pharmaceutical Biotechnology, School of Life Sciences, Nanjing University, Nanjing, China; 4 School of Medical and Molecular Biosciences, Centre for Health Technology Faculty of Science, University of Technology, Sydney, NSW, Australia; INSERM, France

## Abstract

**Background and Aims:**

It has been shown that *Faecalibacterium prausnitzii* (*F. prausnitzii*), one of the dominant intestinal bacterial flora, may protect colonic mucosa against the development of inflammation and subsequent inflammatory bowel disease (IBD), with the underlying mechanisms being unclear.

**Methods:**

The impacts of *F. prausnitzii* and its metabolites on IL-23/Th17/IL-17 pathway markers were determined in human monocytes and a rat model of colitis induced by 2,4,6-trinitrobenzene sulfonic acid. *F. prausnitzii* and its culture medium (containing complete metabolites) were used to treat the rats *in vivo*, as well as rat splenocytes and human monocytes *in vitro*. Inflammatory cytokines were measured in colon tissue, plasma and cell culture medium.

**Results:**

The culture supernatant of *F. prausnitzii* increased plasma anti-Th17 cytokines (IL-10 and IL-12)and suppressed IL-17 levels in both plasma and colonic mucosa, with ameliorated colonic colitis lesions. This inhibition of IL-17 release has also been observed in both rat splenocytes and human venous monocytes *in vitro*. The culture supernatant of *F. prausnitzii* also suppressed Th17 cell differentiation induced by cytokines (TGF-ß and IL-6) and bone marrow-derived dendritic cells (BMDCs) *in vitro*. The metabolites of *F. prausnitzii* in the culture supernatant exert a stronger anti-inflammatory effect than the bacterium itself. *F. prausnitzii* protected the colon mucosa against the development of IBD by its metabolites, suggesting a promising potential for the use of *F. prausnitzii* and its metabolic products in the treatment of IBD.

## Introduction

Inflammatory bowel diseases (IBD), including ulcerative colitis (UC) and Crohn's disease (CD), are characterized by recurrent inflammation in the gastrointestinal tract. The etiology of IBD remains uncertain to date. It has been hypothesized that an undesired intestinal mucosal immune response to luminal contents (e.g., food and bacteria) contributes to the onset of IBD in genetically susceptible individuals.

A number of studies have suggested that the IL-23/Th17/IL-17 pathway plays an important role in the pathogenesis of IBD. Upon co-stimulation by IL-6 and transforming growth factor-beta (TGF-β), native T cells are differentiated into Th17 cells, which release the transcription factor retinoid-related orphan nuclear receptor (RORγt) and Th17 specific cytokines, such as IL-17, IL-17F, and IL-22 [1]. IL-17 released by Th17 cells in turn induces the expression and release of matrix metalloproteases, chemokines and proinflammatory cytokines (eg. TNF-α and IL-6) to mediate monocyte infiltration into the intestinal tissues resulting in tissue damage [2]. IL-17 is also involved in the regulation of neutrophil proliferation, maturation, and chemotaxis [2]. Previous studies have found that IL-17 levels are increased in both serum and colonic mucosa of patients with IBD, compared to those with infectious or ischemic colitis. IL-23 is produced by activated dendritic cells (DCs), which are an essential upstream regulator of Th17 cells, to keep Th17 cells active and functioning [3]. On the other hand, members of the IL-12 cytokine family, such as IL-12 and IL-27, have anti-IL-17 characteristics, which can induce the expression of T box in the T cells (Tbet) and suppress the differentiation and functions of Th17 cells [4], [5].

UC is commonly induced by 2,4,6-trinitrobenzene sulfonic acid (TNBS) in a rat model, which displays similar pathological changes in colorectal tissue as those observed in patients with colorectal colitis. The lesions include mucosal hemorrhages, tissue apoptosis, crypt abscesses, neutrophil infiltration, and increased Th17 cell infiltration in the lesion area [6]. Interestingly, IL-17 receptor deficient mice were less likely to develop colitis after TNBS administration [6]. This may be due to high circulating levels of IFN-γ linked to IL-17 receptor deficiency, which can protect mice from developing an IBD-like phenotype [6]. Furthermore, the administration of either anti-IL-17 or anti-IL-23 antibodies can significantly ameliorate intestinal inflammation in animal models of CD and UC [7], [8]. These findings highlight the critical role of IL-17 in the pathogenesis of IBD-like injury.

Intestinal floral homeostasis has been suggested to contribute to the protective mechanism of the intestinal mucosa against the development of chronic inflammation, including those linked to IBD [9]. The *Faecalibacterium prausnitzii* (*F. prausnitzii*) bacterium accounts for 7% of total fecal bacteria and is one of the predominant bacterial groups in human feces [10]. *F. prausnitzii* metabolizes intestinal lactate to produce butyric acid, which is the main source of generating ATP for the intestinal epithelium [11], [12]. Decreased gut levels of *F. prausnitzii* can result in an ATP shortage in the epithelial cells, thereafter weakening the capacity of self-defense against inflammatory reactions [12]. Butyrate, *per se* has been shown to possess anti-inflammatory properties [13], which may also contribute to the anti-inflammatory effect of *F. prausnitzii* [14].

In patients with IBD, the concentrations of both fecal *F. prausnitzii* and butyric acid were significantly lower compared to the healthy controls [15], [16]. Although fecal bacteria cannot accurately represent the segmental distribution of the colonic mucosa-associated bacteria (MAB), this evidence still suggests that *F. prausnitzii* may be vital in the host defense against the development of IBD [17]. Indeed, in patients with IBD, probiotics supplementation has been shown to significantly reduce colonial mucosal inflammation and ameliorate IBD-related symptoms [14], [18], [19], [20]. The mechanisms may involve the inhibition of pro-inflammatory cytokines (e.g., IL-12 and TNF-α) and the stimulation of anti-inflammatory cytokine secretion (e.g., IL-10) [14], [18], [19], [20], whereas its impact on the IL-23/Th17/IL-17 pathway has not been examined. Nevertheless, the supplementation of *F. prausnitzii* may change the enteric microbiotic homeostasis in patients to improve IBD-related lesions and symptoms.

The hypothesis of this study is that the culture supernatants of *F. prausnitzii* (containing the complete metabolites of *F. prausnitzii*) can ameliorate the development of colorectal UC in rats by inhibiting the differentiation and proliferation of Th17 cells. In this study, the impacts of *F. prausnitzii* and its culture supernatants on the development of UC in Sprague Dawley (SD) rats administered TNBS were determined, along with the effect on the differentiation and cytokine release of Th17 cells from both rat splenocytes and human blood monocytes *in vitro*. The probiotic *Bifidobacterium longum* (*B. longum*) was used as a positive control, which has been shown to inhibit IL-17 release and colitis formation in mice [21].

## Materials and Methods

### 2.1. Ethics approval

Both human and animal experiments were approved by the Ethical Committee of Medical Research, Nanjing Drum Tower Hospital, Affiliated Hospital of Nanjing University Medical School. All healthy participants gave informed consent for human blood cell collection.

### 2.2. Bacterial culture

*F. prausnitzii* ATCC27766 (ATCC, Manassas, VA, USA) was cultured in LYHBHI medium [Brain–heart infusion (37 g/L, BD, Franklin Lakes, NJ, USA), yeast extract (5 g/L, Oxoid, Cambridge, UK), hemin (5 mg/L, Sigma, St. Louis, MO, USA)], supplied with cellobiose (1 g/L, Sigma, St. Louis, MO, USA), maltose (1 g/L, Amresco, Solon, OH, USA), and cysteine (0.5 g/L, Sigma, St. Louis, MO, USA). *Bifidobacterium longum* (Shanghai Sine Pharmaceutical Co., Ltd., Shanghai, China) was cultured in medium containing trypticase (20 g/L Double Spin Microbiological Products Factory, Beijing, China), yeast extract (10 g/L, Oxford, Cambridge, UK), glucose (10 g/L, Sigma, St. Louis, MO, USA), hydrochloric acid cysteine (0.5 g/L, Sigma, St. Louis, MO, USA), dipotassium hydrogen phosphate (0.04 g/L), potassium dihydrogen phosphate (0.04 g/L), calcium chloride (0.008 g/L), and magnesium sulfate heptahydrate (0.008 g/L). Bacteria were cultured in an anaerobic incubator (97% CO_2_ and 3% H_2_, 37°C). The number of live bacteria (colony-forming units, CFU) was determined according to the absorbance at 600 nm (A600). Bacteria were cultured until a stationary phase and then collected with the supernatant lyophilized. Bacterial cells were then washed and suspended at 1×10^9^ CFU/mL in PBS for *in vivo* and *in vitro* studies. For the *in vitro* study, the lyophilized supernatant of *F. prausnitzii* was prepared as 1×, 2×, 5×, and 10× concentrations prior to use. The sodium butyrate stock solution was 5.834 µmol/ml. All experiments used lyophilized supernatant unless stated otherwise.

### 2.3. Modeling of colorectal colitis in rats and treatment

The rats (SD, 6 weeks, males) were obtained from the Animal Center, Nanjing Drum Tower Hospital (Nanjing, China) and the *in vivo* experiment was performed in the same facility. The rats were treated daily with different reagents (gavage, catheter 1.2 mm in diameter), including PBS, *F. prausnitzii* (1×10^9^ CFU/rat/day), 1× and 5× concentrated supernatant of *F. prausnitzii*, and *B. longum* (1×10^9^ CFU/rat/day, Beijing, Technology Co., Ltd., Beijing, China) for 7 consecutive days. On day 6 of the oral treatment, colitis was induced by TNBS as previously described [22]. Briefly, the TNBS (Sigma, St. Louis, MO, USA) solution was slowly administered in the colon (80 mg/kg) via a 4.7 mm-diameter catheter (Jiangyin Guoguang Rubber Product Co., Ltd., Jiangyin, China). The control was administered with vehicle. On day 8, the rats were weighed and killed using ether exposure. Colons were removed, measured, and fixed in 4% formalin for later histology examination. The colon specimens were then stained with hematoxylin and eosin, and the lesion was analyzed using Neurath Scoring criteria as previously described [23] (briefly, 0  =  no inflammation; 1  =  very low level of leucocyte infiltration; 2  =  low level of leucocyte infiltration; 3  =  high level of leucocyte infiltration, high vascular density, thickening of the colon wall and 4 =  transmural leucocyte infiltrations, loss of goblet cells, high vascular density, and thickening of the colon wall).

### 2.4. Peripheral blood mononuclear cell (PBMC) culture

PBMCs were isolated from the venous blood of healthy donors as previously described [24]. Briefly, after Ficoll-Isopaue density gradient centrifugation (Ficoll-Paque, MP Biomedicals, Carlsbad, USA), mononuclear cells were collected and washed in RPMI-1640 medium (Invitrogen, NY, USA) supplemented with 10% fetal bovine serum (FBS; Invitrogen, NY, USA), L-glutamine (2 mmol/L), penicillin (100 U/ml) and streptomycin (100 U/ml). Subsequently, cells were suspended in complete medium (2×106 cells/ml) and seeded in a 24-well plate (2×106 cells/well). PBMCs were then treated with 1×, 2×, 5×, and 10× concentrated supernatant of F. prausnitzii. After 24 h, the culture supernatant of the PBMCs was collected and stored at −80°C for cytokine analysis.

### 2.5. Primary splenocyte and bone marrow-derived dendritic cell (BMDC) culture

Untreated 6-week-old SD rats were sacrificed by cervical dislocation after ether exposure. Cells in the spleen were harvested and prepared as a single cell suspension in complete medium in a 24-well plate (1×10^7^ cells/well). In the control group, splenocytes were incubated alone or with recombinant human TGF-ß (2 ng/ml, Peprotech, Rocky Hill, NJ, USA) and recombinant rat IL-6 (20 ng/ml, Peprotech, Rocky Hill, NJ, USA) at 37°C for 72 h. For the experimental groups, in addition to TGF-ß and IL-6, cells were treated with culture supernatant of *F. prausnitzii*, sodium butyrate (0.05834 µmol/well), denatured *F. prausnitzii* and *B. longum* bacteria (1× 10^7^ CFU/well). Each group treatment was repeated four times. After 72 h, the supernatant of cultured splenocytes was collected and stored at −20°C for further analysis [21].

Immature BMDCs were isolated using a protocol modified from previous publications [25], [26]. Briefly, both ends of the rat (6 weeks) femur and tibia bones were cut, and the bone marrow flushed out using RPMI-1640 medium (Invitrogen, NY, USA). The tissue was suspended and passed through a nylon mesh to remove debris. The red cells were removed with red blood cell lysis buffer (KeyGEN, Nanjing, China). Bone marrow cells (1×10^6^ cells) were then cultured in 1 ml complete medium for 7 d in the presence of recombinant rat IL-4 (10 ng/ml, Peprotech, Rocky Hill, NJ, USA) and recombinant rat granulocyte-macrophage colony-stimulating factor (GM-CSF, 10 ng/ml, Peprotech, Rocky Hill, NJ, USA). At day 7, immature BMDCs were resuspended in complete medium for maturation. Mature BMDCs (1×10^6^ cells/well) were incubated with LPS (1 µg/ml, from *E. coli*, Sigma-Aldrich, St. Louis, MO, USA) for 12 h and then treated with culture supernatant of *F. prausnitzii* (1 µl), sodium butyrate solution (0.005834 µmol) and denatured *F. prausnitzii* or *longum* bacteria (1×10^6^ CFU/well, BMDCs: bacteria  =  1:1) at 37°C for another 12 h. PBS treated BMDCs were used as a control. Each group treatment was repeated four times. The supernatant was collected and stored at −20°C.

In another cohort, the BMDC cells were incubated with LPS for 12 h before splenocytes (1×10^7^ cells) were added to each well (BMDCs: splenocytes  =  1:10). Subsequently, the cell mixture was treated with the culture supernatant of *F. prausnitzii* (1 µl), sodium butyrate solution (0.005834 µmol), denatured *F. prausnitzii* or *B. longum* bacteria (1×10^6^ CFU/well, 1 µl) and incubated for 72 h. The cell mixture treated with PBS was used as a negative control. Each group was repeated four times. The supernatant was collected at 72 h and stored at −20°C.

### 2.6. ELISA assay

Cytokines (IL-10, IL-17A, IL-12 p70, and IL-23) were measured using a commercially available ELISA kit (Bender, Vienna, Austria; SABC, Wuhan, China) according to the manufacturers' instructions.

### 2.7. Immunohistochemistry

Colon specimens were fixed in 4% formalin and embedded in paraffin. Sections were boiled in Tris-EDTA buffer (pH 9.0) for 20 min and cooled at room temperature. Sections were then incubated with rabbit anti-rat IL17 antibodies (Abcam, Cambridge, UK) at 4°C for 24 h and then with the corresponding secondary antibody (Zsbio, Beijing, China) at 37°C for 30 min. The sections were then treated with immunoperoxidase using the DAB kit (ZLI-9033 system, Zsbio, Beijing, China). Sections were scored in a blind manner using a protocol modified from a previous publication [27].

### 2.8. SCFA assay

Fresh rat fecal samples were collected from home cages, weighed, and stored at −80°C. Fecal samples were mixed with distilled water and centrifuged. The supernatant was removed, filtered, and mixed with ether and sulfuric acid. After centrifugation, the ether layer was collected and measured in an Agilent 6890N Gas Chromatograph Machine for SCFA (acetic acid, propionic acid, isobutyric acid, butyric acid and, isovaleric acid) concentrations.

### 2.9. Statistics

Data were expressed as the mean ± standard deviation of the mean (SD). The data were analyzed with a one-way ANOVA followed by a post hoc Duncan test (SPSS 17.0, SPSS Inc., Chicago, USA). *P* <0.05 was considered significant.

## Results

### 3.1. Body weight and colon histology

**Figure 1:**
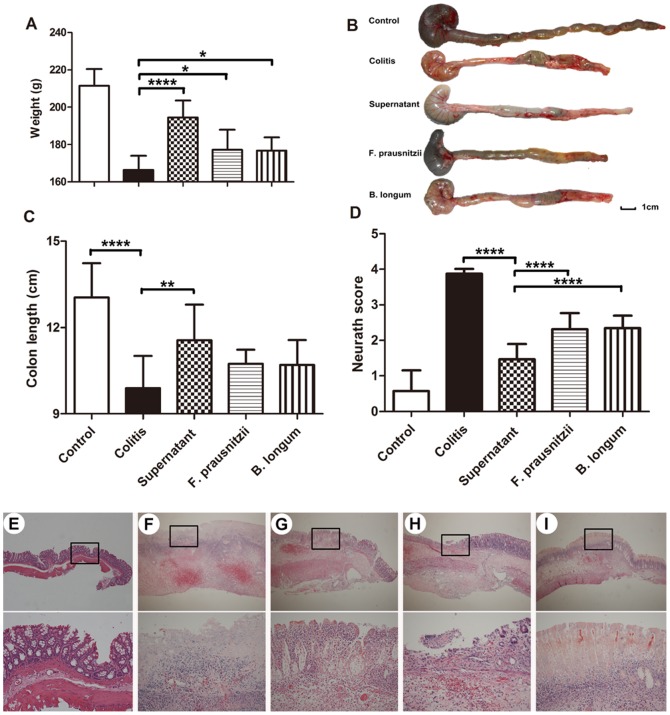
Growth and colonic lesion in rats. Body weight change (A). Colon length (B, C). Colon Neurath score (D). Representative images of rat colonic mucosa (E–I), control group (E); colitis group (F); supernatant group (G); *F. prausnitzii* group (H); *B. longum* group (I). Upper and lower panel magnifications are ×40 and ×200, respectively. Data are the mean ± SD. *n*  =  7–8. *P <0.05; **P <0.01; ****P <0.001.

At the end of the experiment, compared with the control group, the rats in the colitis-TNBS treated group had significantly smaller weight gain, as well as a shorter colon and higher Neurath scores (*P* <0.05; Figures 1A–F). The culture supernatant of *F. prausnitzii* significantly ameliorated the weight loss, reduction in colon length and high Neurath scores observed in rats in the colitis group (*P* <0.05; supernatant vs. colitis groups; Figures 1A–D, F–G). However, the treatments with either *F. prausnitzii* bacteria or *B. longum* only significantly reduced the Neurath score and body weight gain (*P* <0.05, compared with the colitis group; Figures 1A–D), without affecting colon length.

Histological examination showed that the rats in the colitis group developed extensive ulceration in their colons, which consisted of coagulative necrosis within the lesion surrounded by large numbers of neutrophils and some mononuclear cells. Necrosis lesions penetrated deeply into the muscularis propria. *F.prausnitzii* and culture supernatant-treated rats only displayed mild mucosal and/or submucosal inflammation with a relatively low level of neutrophil infiltration and mild edema (Figures 1E–I).

### 3.2. Cytokine and intestinal fatty acid levels in rats

**Figure 2:**
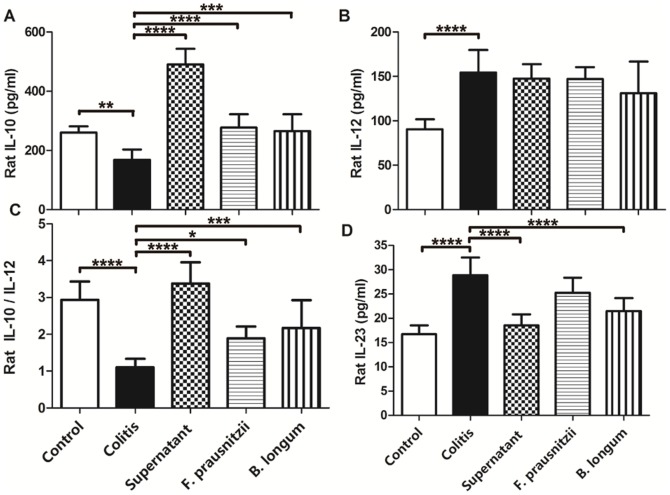
Plasma cytokine concentrations in rats. IL-10 (A). IL-12 (B). IL-10/IL-12 ratio (C). IL-23 (D). Data are the mean ± SD. *n*  =  7–8. *P <0.05; ***, *P* <0.003; ****, *P* <0.001.

**Figure 3:**
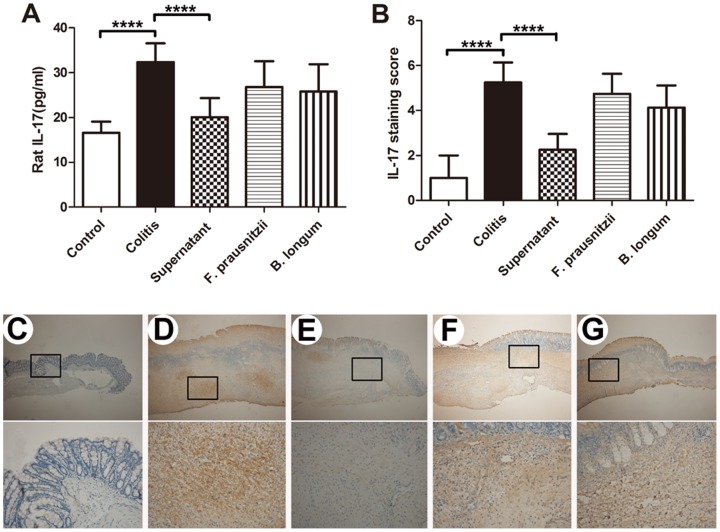
IL-17 protein expression in rat plasma and colon. Plasma IL-17 concentrations in the rat (A). IL-17 protein immunohistochemical staining in rat colon (B). *n*  =  7–8. Data are the mean ± SD. ****P <0.001. Representative immunohistochemical staining of IL-17 in rat colon mucosa in the control (C), colitis (D), supernatant (E), *F. prausnitzii* (F), and *B. longum* (G) groups. Upper and lower panel magnifications are ×40 and ×200, respectively.

Plasma cytokines, such as IL-12, IL-10,the IL-10/IL-12 ratio, and IL-23, can be used to assess the systemic level of inflammation [24]. Compared with the control group, plasma levels of IL-12 and IL-23 in the colitis group were significantly higher (*P* <0.05; Figures 2Band 2D), and the IL-10 levels and IL-10/IL-12 ratio in the colitis group were significantly lower (Figures 2A and 2C). Plasma levels of IL-10 and the IL-10/IL-12 ratio in the supernatant group were greater compared with those in the colitis group (*P* <0.05; Figures 2A and 2D). Plasma IL-23 levels were only reduced by the culture supernatant of *F. prausnitzii* and *B. longum* treatments compared with those in the colitis group (Figure 2D). In rats, IL-17 can be detected in both plasma and colon tissue. Compared with the control group, IL-17 levels in both plasma and colon tissue were greater in the colitis group (*P* <0.05; Figures 3A, 3B and 3C–G), and were only decreased by the culture supernatant of *F. prausnitzii* (*P* <0.05; colitis vs. supernatant groups; Figures 3A, 3B and 3C–G).

**Figure 4:**
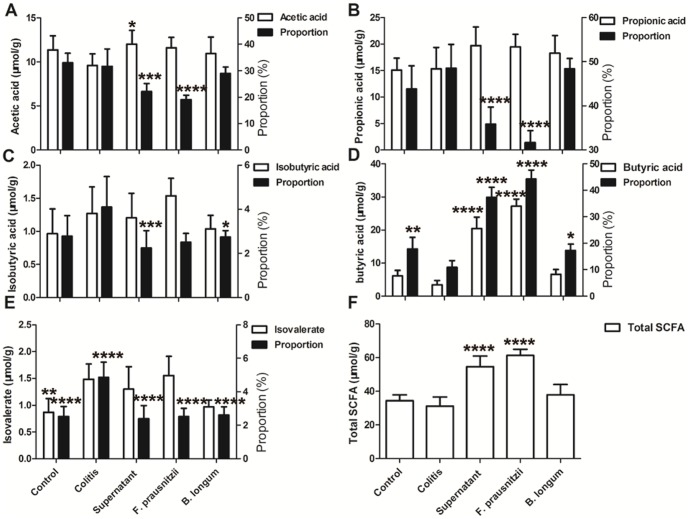
Fecal SCFA concentrations and proportion of total SCFA content in rats. (A) Fecal acetic acid concentration and percentage as total SCFA content. (B) Fecal propionic acid concentration and percentage of total SCFA content. (C) Fecal isobutyric acid concentration and percentage of total SCFA content. (D) Fecal butyric acid concentration and percentage of total SCFA content. (E) Fecal isovalerate concentration and percentage of total SCFA content. (F) Fecal total SCFA content. Data are the mean ± SD. *n*  =  7–8. *, *P* <0.05; **, *P* <0.01; ***P <0.003; ****P <0.001, compared with the colitis group.

In the colitis rats, the net concentrations of butyric acid, total fecal short chain fatty acid (SCFA) and the percentage of butyric acid of the total SCFA was significantly lower than that in controls (*P* <0.05; Figure 4D and 4F). Compared with the colitis group, fecal concentrations of butyric acid and total SCFA, as well as the percentage of butyric acid in the supernatant group and *F. prausnitzii* group, were higher (*P* <0.05; Figures 4D and 4F). The percentages of the other SCFAs of the supernatant group were significantly lower compared with the colitis group (Figures 4A, 4B and 4C–E). Of note, the SCFA concentrations in the reconstituted culture supernatant (1×, 5×) used for this study are listed in Table 1.

**Table 1:** Short-chain fatty acids (*SCFAs*) in fresh cultured and concentrated supernatants.

Culture medium	Acetic acid	Propionic acid	Isobutyric acid	Butyric acid	Isovalerate
*F. prausnitzii* fresh (µmol/ml)	3.9±0.85	0.22±0.04	0.12±0.01	33.18±6.45	0.07±0.01
*F. prausnitzii* 1× (µmol/ml)	3.46±0.2	1.11±0.06	0.27±0.01	5.83±0.24	0.56±0.02
*F. prausnitzii* 5× (µmol/ml)	17.29±1	5.57±0.28	1.35±0.07	29.17±1.22	2.81±0.11
*B. longum* fresh (µmol/ml)	1.38±0.06	2.21±0.03	0.08±0.01	0.9±0.03	0.06±0

Data are presented as the mean ± SD. *n*  =  3.

*F. prausnitzii* fresh, fresh culture supernatant of *F. prausnitzii*(1×10^8^ CFU/ml). *F. prausnitzii* 1×, one time reconstituted culture supernatant of *F. prausnitzii*. *F. prausnitzii* 5×, five times reconstituted culture supernatant of *F. prausnitzii*. *B. longum* fresh, fresh culture supernatant of *B. longum*(1×10^8^ CFU/ml).

### 3.3. Anti-inflammatory cytokine release in human peripheral blood mononuclear cells (PBMCs) in vitro

**Figure 5:**
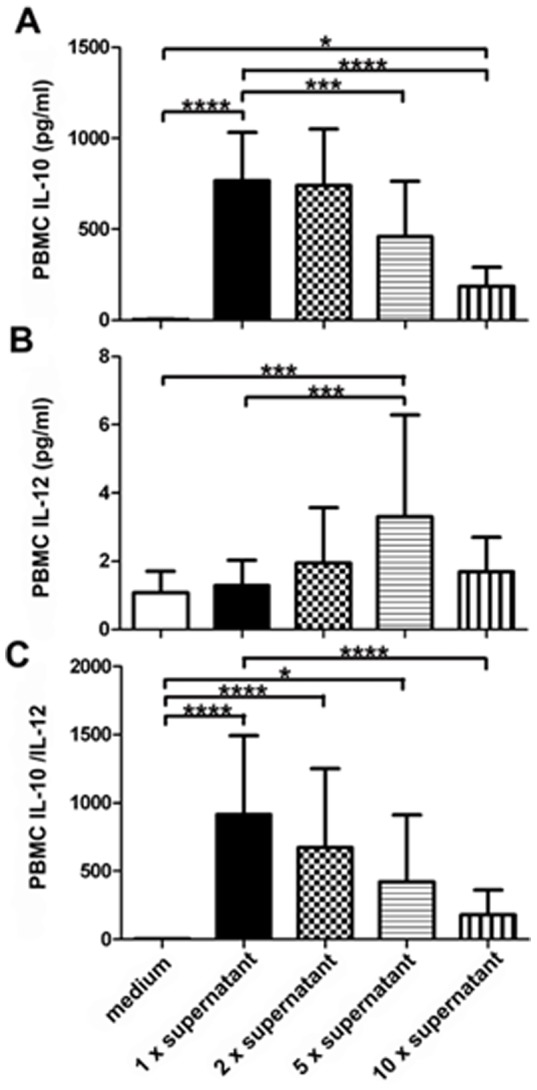
IL-10 and IL-12 release from healthy human PBMCs. IL-10 (A). IL-12 (B). IL-10/IL-12 ratio (C). Data are the mean ± SD; *n*  =  10–14. *, P <0.05; **, P <0.01; ***, P <0.003; ****, P <0.001. Medium, *F. prausnitzii* cell culture medium. 1× supernatant, one time concentrated supernatant. 2× supernatant, two times concentrated supernatant. 5× supernatant, five times concentrated supernatant. 10× supernatant, ten times concentrated supernatant.

PBMCs were sourced from the venous blood of healthy adults. The effects of culture supernatant of *F. prausnitzii* on IL-10, IL-12 and the IL10/IL-12 ratio release displayed a dose-dependent effect, with the greatest impact observed at the lowest concentration (Figures 5A, 5B and 5C).

### 3.4. Th17 cell differentiation in vitro

**Figure 6:**
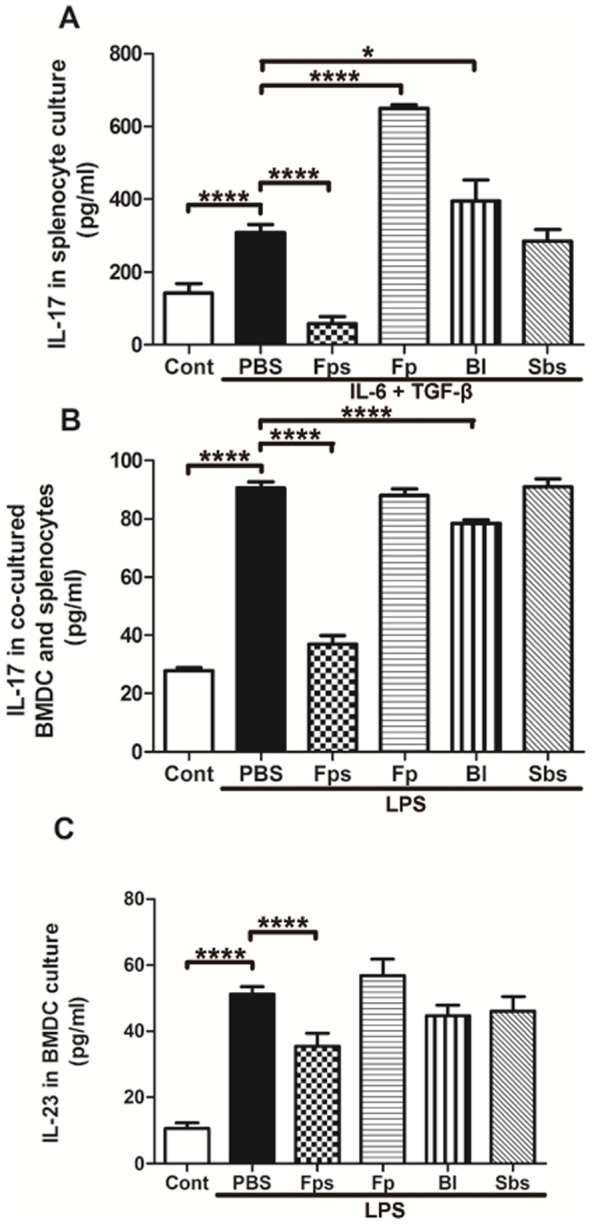
IL-17 and IL-23 release *in vitro*. IL-17 release from rat splenocytes *in vitro* (A). IL-17 release from co-cultured BMDCs and splenocytes *in vitro* (B). IL-23 released from the BMDCs with LPS stimulation (C). Data are the mean ± SD; *n*  =  4. *, P <0.05; ****, P <0.001. Cont, control group. PBS, PBS group. Fps, supernatant group. Fp, *F. prausnitzii* group. Bl, *B. longum* group. Sbs, sodium butyrate group.

IL-6 and TGF-β can stimulate native T cells to differentiate into Th17 cells, which in turn produce and secrete IL-17 [1]. *In vitro*, IL-17 release from rat splenocytes was significantly increased by IL-6 and TGF-β compared with the non-stimulated control group (*P* <0.05; PBS vs. control; Figure 6A). Compared with the PBS group, IL-17 secretion was significantly lower in cells cultured with the supernatant of *F. prausnitzii* (*P* <0.05; Fps vs. PBS; Figure 6A); whereas IL-17 secretions were greater in the splenocytes co-cultured with *F. prausnitzii* (Fp) or *B. longum* (Bl), compared with the PBS group (*P* <0.05; Figure 6A). Butyric acid, a type of anti-inflammatory substance, was highly present in the supernatant of *F. prausnitzii* (Table 1). To verify its role in Th17 cell differentiation, we used the same sodium butyrate concentration as in the supernatant of *F. prausnitzii* and found that IL-17 secretion was not significantly changed when splenocytes were cultured with sodium butyrate compared with the PBS group (*P* <0.05; Figure 6A).

Immature bone marrow dendritic cells (BMDCs) can be activated by lipopolysaccharide (LPS) to secrete IL-23 [28], [29], which plays an important role in maintaining the stability and function of Th17 cells [3]. *In vitro*, IL-23 secretion in the immature BMDCs was significantly increased by LPS compared with the non-stimulated control group (*P* <0.05; PBS vs. control; Figure 6C). Compared with the PBS group, IL-23 secretion was significantly lower only in cells cultured with the supernatant of *F. prausnitzii* (*P* <0.05; Fps vs. PBS; Figure 6C). When splenocytes and BMDCs were co-cultured, the IL-17 level was significantly increased by LPS in the PBS group, compared with the non-stimulated control group (*P* <0.05; Figure 6B). In LPS treated cells, those incubated with the supernatant (Fps) and *B. longum* (Bl) released significantly less IL-17 into the culture medium (*P* <0.05; Figure 6B).

## Discussion

The major findings in this study are that the metabolites of *F. prausnitzii* in the culture supernatant displayed potent effects that ameliorated colitis lesions in a rat model with similar features to those in patients with IBD. The proposed mechanisms resulting from this study are: 1) the inhibition of inflammatory cytokine (IL-17) release, and 2) the changes of the ratio in inflammatory cytokines (i.e., IL-10/IL-12) released by monocytes.

The causes of IBD are multifactorial. Recently, it has been recognized that disturbed intestinal bacterial homeostasis may also contribute to the onset and recurrence of IBD. *Firmicutes* and *Bacteroidetes* are the dominant bacterial strains in human feces [10]. Several studies confirmed that a reduction of *F. prausnitzii*, a well-known butyrate-producing bacterium, in IBD patients showed an inverse correlation with disease activity [15], [16]. *F. prausnitzii*'s metabolic products have been shown to possess anti-inflammatory properties and it has also been suggested that a sudden decrease in *F. prausnitzii* in the mucosa may play a role in the recurrence of IBD [14]. Subsequent studies have suggested that the protective effect of *F. prausnitzii* is due to pro-inflammatory cytokine inhibition and Treg promotion [20], [30]. However, this study found that the protective effect of *F. prausnitzii* is partly through Th17 cell inhibition. Therefore, *F. prausnitzii* could be a potential alternative for the treatment of IBD.

Both IL-10 and IL-12 can suppress Th17 cell differentiation by activating its inhibitor Th1 cells [4]. The ratio of IL-10/IL-12 can be used to assess the anti-inflammatory effects of bacteria *in vivo* and *in vitro* [24]. In the current study, the culture supernatant of *F. prausnitzii* could increase both systemic and local inflammatory cytokines in rats, due to increased levels of IL-10, as well as the IL-10/IL-12 ratio in the plasma and colonic tissues. It seems that the culture supernatant of *F. prausnitzii* exerted stronger anti-inflammatory effects than *F. prausnitzii* and *B.infantis*. The effects of the culture supernatant of *F. prausnitzii* were consistent between the *in vivo* rat and *in vitro* human PBMC studies [30]. This indicates that the chemicals released by the *F. prausnitzii* play a key role in the anti-inflammatory effects of *F. prausnitzii*, which did reduce the colorectal inflammatory lesion induced by TNBS in rats. Interestingly, probiotic *B.infantis* was shown to be able to protect individuals against the development of UC in the clinic, and therefore, in this study, it was used as a positive control [21]. however,*B. infantis* treatment did not have an impact on IL-10 and IL-12, suggesting that the suppressive effects of *B.infantis* on the development of colitis occur via different pathways other than the IL-10/IL-12 ratio.

IL-23 acts as the upstream regulator of Th17 cells, which is criticalfor maintaining the stability and activation of Th17 cells [3]. In this study, circulating IL-23 and IL-17 were both significantly increased by TNBS treatment, suggesting their involvement in the onset of UC. IL-23 was markedly suppressed upon treatment of the culture supernatant of *F. prausnitzii*. This suggests that it could be chemicals with short half-lives released by *F. prausnitzii* that suppressed IL-23 release. In this study, IL-17 levels in the plasma and colonic mucosa, as well as IL-23 levels in the plasma, were significantly increased in rats with colitis, which is similar to patients with CD and UC [8], [31], [32]. Both IL-23 and IL-17 levels were significantly lower in the culture supernatant treated rats, in the face of less developed UC. This suggests that the IL-23/Th17/IL-17 pathway is an effective target for the treatment of inflammatory colitis. The culture supernatant of *F. prausnitzii* still exerted stronger effects than the bacteria *F. prausnitzii* and *B.infantis* in reducing IL-17 levels. In the short term, this may be due to the bacteria being unable to produce as many metabolites as those accumulated in the culture supernatant over time and therefore having less of an effect in suppressing IL-17. The *in vitro* experiments in the rat splenocytes and BMDC further confirmed this finding in the *in vivo* rat data. When splenocytes differentiated into Th17 cells *in vitro*, they showed a similar cytokine release profile as those observed in the colitis rats. The culture supernatant of *F. prausnitzii* could suppress the secretion of IL-17 by the splenocytes when they were co-cultured with BMDCs.

Butyric acid is one of the metabolites produced by *F. prausnitzii* [11], which is an important energy source for the intestinal epithelial cells [12] and has an inverse correlation with disease activity [15], [17]. Using gas chromatography, we found that *F. prausnitzii* produced large amounts of butyric acid in the culture supernatant, which could contribute to the significant fecal butyric acid content and its concentration in *F. prausnitzii* and the culture supernatant of *F. prausnitzii*-administered rats, suggesting that the bacterium itself is essential for maintaining high levels of butyric acid in the intestinal lumen. The increase in fecal butyric acid concentration is causally related to ameliorated tissue lesions, suggesting that butyric acid may be involved in the prevention of TNBS-induced colitis in the rat. Indeed, butyric acid has been shown to have an anti-inflammatory effect *in vitro* [33], [34]. This study also observed that butyric acid suppressed the inflammatory cytokine release from rat splenocytes *in vitro*, although no significantly difference was observed. This result suggests that other unknown metabolites, rather than butyric acid, played a key role in reducing the development of IBD.

**Figure 7:**
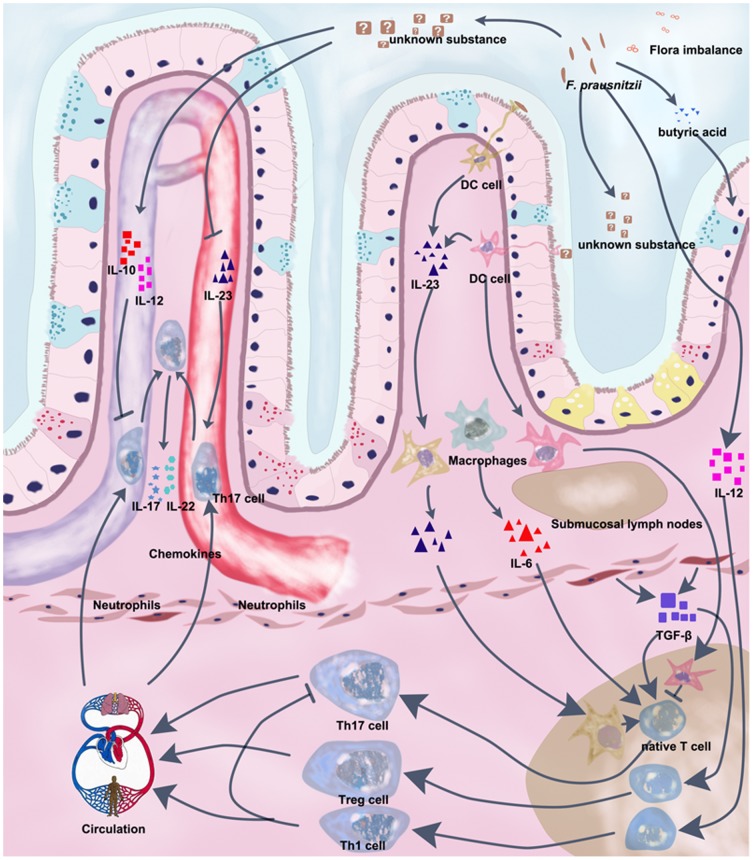
Proposed working mechanism of the anti-inflammatory effect of *F. prausnitzii* metabolic products. The *F. prausnitzii* metabolic products act via mechanisms on the IL-23/Th17/IL-17 axis. The *F. prausnitzii* metabolic products inhibit Th17 activity by inhibiting IL-23 secretion from the DCs and reduce the number of Th17 cells by inducing the anti-inflammatory cytokines. → stimulation; -| inhibition.

In summary, this study showed that metabolites of *F. prausnitzii* exerted significant anti-inflammatory effects in colorectal colitis in rats (proposed working model in Figure 7), which ameliorated the tissue lesions. As similar anti-inflammatory effects have been observed in human monocytes, using *F. prausnitzii* metabolic products is an option for further investigations on potential treatments for IBD.
